# Juxtaglomerular cell tumor diagnosed preoperatively by renal tumor biopsy

**DOI:** 10.1002/iju5.12283

**Published:** 2021-04-07

**Authors:** Takashi Ueda, Yukiko Morinaga, Kai Inoue, Sojiro Hirano, Hiroki Matsubara, Fumiya Hongo

**Affiliations:** ^1^ Department of Urology Kyoto Prefectural University of Medicine Kyoto‐City Kyoto Japan; ^2^ Department of Pathology Kyoto Prefectural University of Medicine Kyoto‐City Kyoto Japan; ^3^ Department of Urology Kyoto Yamashiro General Medical Center Kizugawa‐City Kyoto Japan

**Keywords:** hypertension, hypokalemia, juxtaglomerular cell tumor, laparoscopic partial nephrectomy, renal tumor biopsy

## Abstract

**Introduction:**

Diagnosis of small renal tumor from imaging analysis is limited. We report a case of juxtaglomerular cell tumor diagnosed preoperatively by renal tumor biopsy.

**Case presentation:**

A 17‐year‐old male was urgently hospitalized for acute‐onset congestive heart failure. Radiographic findings revealed a 2‐cm mass lesion, and on renal biopsy, a juxtaglomerular cell tumor was suspected. The juxtaglomerular cell tumor was resected by laparoscopic partial nephrectomy, on suspicion of causing the heart failure. The patient’s clinical symptoms improved drastically postoperatively.

**Conclusion:**

Biopsy may be a promising option for preoperative diagnosis of juxtaglomerular cell tumors.

**Abbreviations & Acronyms**
ALT: alanine aminotransferase
AST: aspartate aminotransferase
CK: cytokeratin
CT: computed tomography
HHF35: muscle actin
JGCT: juxtaglomerular cell tumor
MRI: magnetic resonance imaging
Na: natrium
PAX: paired box
SMA: smooth muscle actin
SOX: SRY‐related HMG box
T‐Bil: total bilirubin
UA: uric acid

**Keynote message**

Some renal tumors may be difficult to diagnose based on radiographic findings. This case report provides valuable insight on the use of renal tumor biopsy for appropriate diagnosis. The JGCTs should be taken into consideration in diagnosis of renal tumors with characteristic symptoms such as hypertension and hypokalemia.

## Introduction

With widespread utilization of CT for screening diseases of patients, frequency of incidental findings of small renal masses is getting higher recently.^1^ Though enhanced CT and MRI are used to presume the histology for decision of treatment courses, there is limitation for presumption from imaging analysis.^2^ Renal tumor biopsy may be a promising technique to diagnose small renal masses.^3^

JGCT is one kind of the diseases which can present small renal mass.^4^ Since most of the JGCT patients show characteristic symptoms, such as hypertension and hypokalemia, due to oversecretion of renin from the tumor, diagnosis of the disease had been made mainly based on the presence of the characteristic symptom and radiographic findings. Here, we present the case of a young male patient, who was preoperatively diagnosed with JGCT using renal tumor biopsy.

## Case presentation

**Fig. 1:**
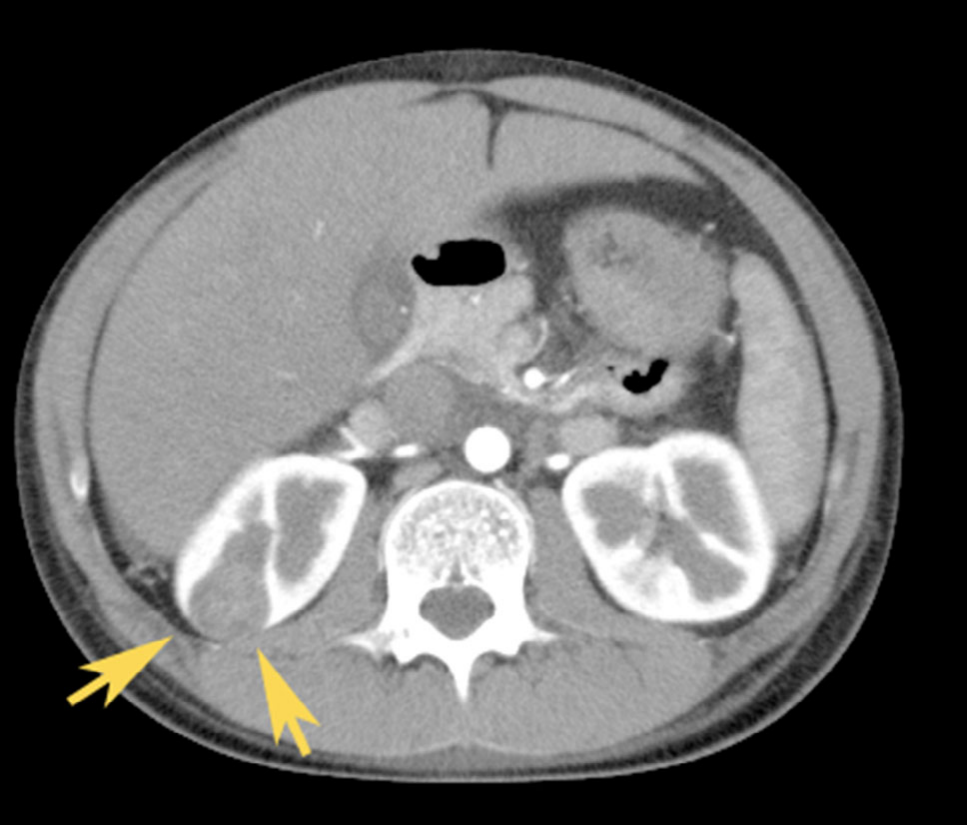
Enhanced CT showing a 2‐cm mass lesion with slight and uneven enhancement in right kidney. Arrows: tumor.

**Fig. 2:**
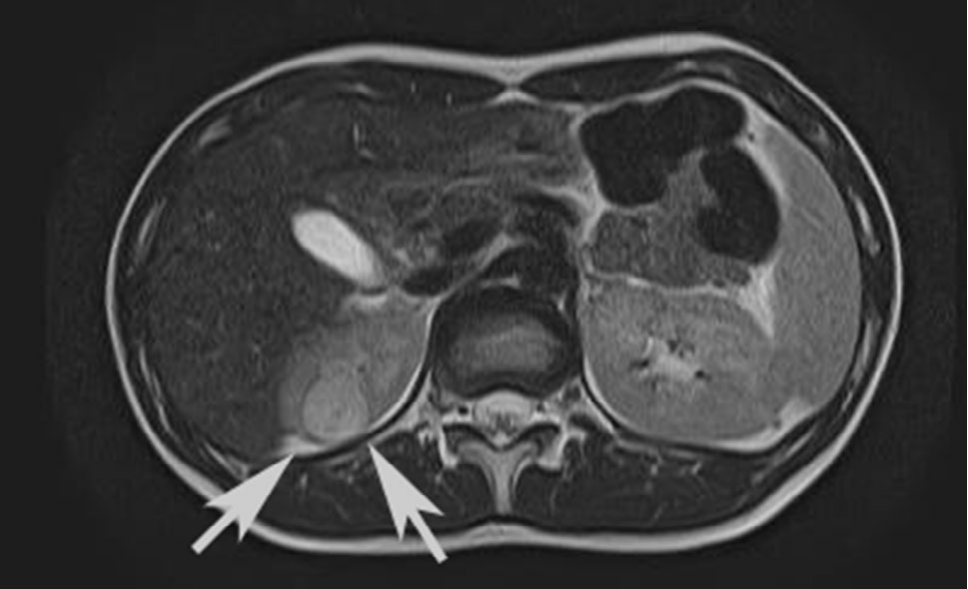
MRI showing a hyperintense lesion on the T2‐weighted image. Arrows: tumor.

**Fig. 3:**
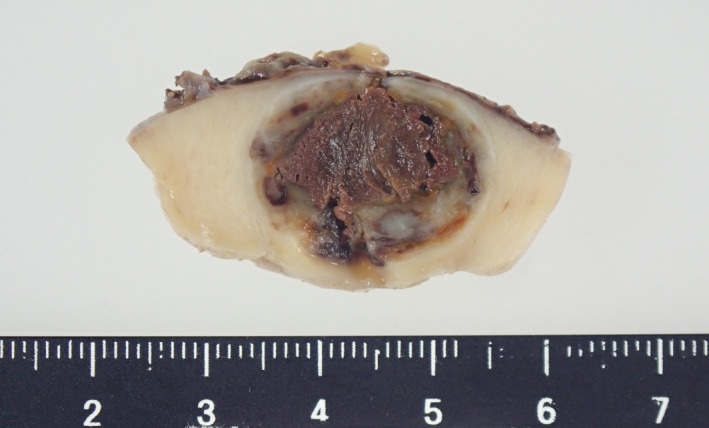
Macroscopic figure of the tumor. The cut surface was grayish‐tan, and prominent hemorrhage and hemosiderin deposition were observed on the inside.

**Fig. 4:**
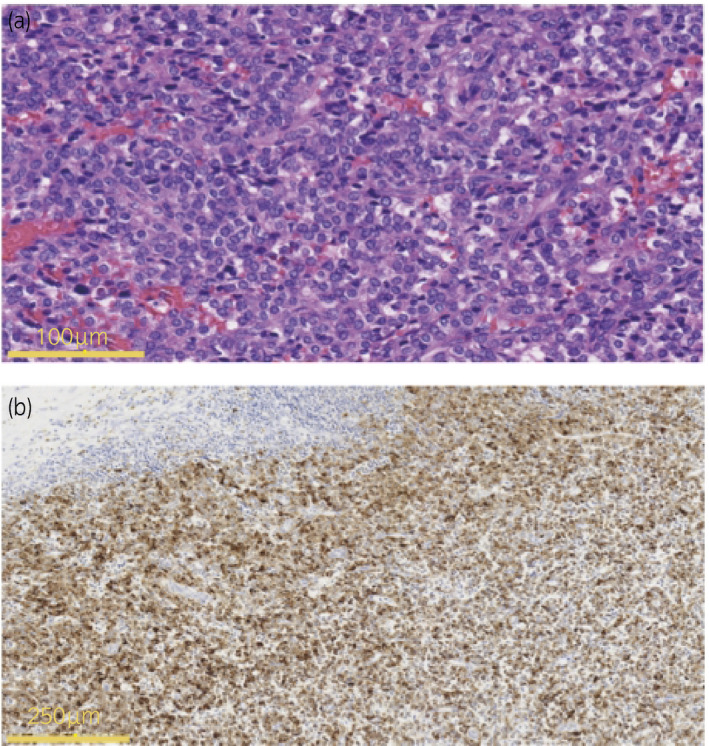
(a) Histological findings in the tumor. The tumor was composed of uniform polygonal cells with small round nuclei and clear or lightly eosinophilic narrow cytoplasm, showing a cord‐like or solid growth pattern. (b) Immunohistochemical analysis. Tumor cells were positive for renin. This finding, combined with CD34 positivity and negativity for CK marker, is consistent with a JGCT.

A 17‐year‐old male visited our hospital because of epigastric abdominal pain. His blood pressure was high (133/104 mmHg) and physical examination revealed leg edema. His laboratory results revealed the following values: T‐Bil: 2.97 mg/dL (normal 0.2–1.2 mg/dL), AST: 576 U/L (normal 8–38 U/L), ALT: 967 U/L (normal 4–44 U/L), UA: 8.7 mg/dL (normal 2.5–6.8 mg/dL), Na (sodium): 128 mEq/L (normal 138–145 mEq/L), and kalium: 3.5 mEq/L (normal 3.6–4.8 mEq/L). Cardiac ultrasound suggested acute‐onset congestive heart failure with a left ventricular ejection fraction of 19%, and the patient was urgently hospitalized for the heart failure treatment. Obstructive coronary artery disease was excluded as a cause by cardiac catheterization. The patient was diagnosed with dilated cardiomyopathy by a myocardial biopsy. A 2‐cm mass lesion with slight, uneven enhancement in the right kidney was incidentally found by enhanced CT (Fig. 1). On MRI, the renal mass was isointense lesion on T1‐weighted image and hyperintense lesion on T2‐weighted image (Fig. 2). Histological diagnosis by renal biopsy revealed a JGCT. At this point, a functional juxtaglomerular tumor was suspected as the cause of the patient’s symptoms such as dilated cardiomyopathy. Preoperative plasma renin level was 4.8 ng/mL (normal 0.3–2.9 ng/dL) and aldosterone level was 165 pg/mL (normal 29.9–159 pg/mL). The renal tumor of the right kidney was partially resected laparoscopically. Grossly, the tumor was a well‐circumscribed nodule with an incomplete fibrous capsule. The cut surface was grayish‐tan, and prominent hemorrhage and hemosiderin deposition were observed on the inside (Fig. 3). Histologically, the tumor was composed of uniform polygonal cells with small round nuclei, and clear or lightly eosinophilic narrow cytoplasm, showing a cord‐like or solid growth pattern (Fig. 4a). Intervening between the tumor cells and abundant capillaries and lymphocyte infiltration was observed. No nuclear pleomorphism or mitotic figures were observed. Immunohistochemically, the tumor cells showed renin (+), CD34 (+), CK‐P (a cocktail of CK AE1/AE3 and PCK26) (−), c‐kit (±; a few), HHF35 (−), desmin (−), SMA (−), calponin (−), CD56 (−), PAX8 (−), and SOX10 (−) (Fig. 4b).

The final diagnosis of the patient was a JGCT based on the histological findings of the resected tissue. Postoperatively, the patient’s symptoms are improved (blood pressure: 123/180 mmHg) with decrease of plasma renin (0.4 ng/mL) and aldosterone (53.7 pg/mL). Other postoperative laboratory results were as follows: T‐Bil: 0.75 mg/dL, AST: 18 U/L, ALT: 15 U/L, UA: 6.7 mg/dL, Na: 139 mEq/L, and kalium: 3.6 mEq/L. Cardiac ultrasound suggested improved cardiac function with a left ventricular ejection fraction of 61.0%.

## Discussion

JGCT is a rare disease which presents renal mass by CT, and about 100 cases have been reported until now.^5^ Since JGCT is more prevalent in young female adults,^6^ several cases during pregnancy have been reported as well.^7^ Most of the JGCT causes oversecretion of renin and as a result of that the patients present with hyperaldosteronism symptom such as hypertension and hypokalemia.^8^ Increased levels of plasma renin and aldosterone and characteristic symptom are diagnostic clue of JGCT.

On dynamic CT, JGCT tumors are not enhanced or only slightly enhanced in the late phase. On MRI, JGCT tumors show isointense or hypointense lesion on T1‐weighed images, and hyperintense lesion on T2‐weighted image,^9^ as in this case.

Histologically, JGCT cells are composed of uniform polygonal cells with small round nuclei and eosinophilic cytoplasm, showing solid growth pattern.^10^ Immunohistochemically, JGCT cells show positive CD34 and renin.^6^ In most of the previously reported cases, surgical resection had been performed. Because the incidence of the disease is common in young adults, partial nephrectomy or ablation is thought to be an appropriate procedure for the patients to spare the nephron. In most cases, clinical presentations, such as hypertension, caused by the tumor have improved after tumor resection. In one case, severe drop of blood pressure after ablation of tumor was reported.^11^ A saline infusion improved the blood pressure in the case.

As described above, preoperative diagnosis of JGCT has been presumed by the clinical presentation, elevation of plasma renin, and radiographic findings in most of the previous cases. There were some cases in which the patients underwent surgery with a clinical diagnosis of renal cell carcinoma, but the pathological diagnosis was that of JGCT.^10^, ^12^ Moreover, it has been reported that other tumors can produce renin, such as desmoplastic small round cell tumors and Wilms tumor.^13^ On the contrary, there is a nonfunctional variant of JGCT, which does not present hyperaldosteronism symptoms. Similarly, in some cases, there has been difficulty in diagnosing JGCT due to the lack of characteristic symptoms, resulting in delayed treatment causing unfortunate outcomes.^14^ Our patient was not suspected to have JGCT until biopsy of the renal tumor was performed, and transplantation of the heart was considered a treatment option for the dilated cardiomyopathy. Recently, renal tumor biopsy is considered to be a safe and beneficial option for diagnosis of patients with renal tumor though there are mild complications such as bleeding and infection.^15^ Therefore, we concluded that biopsy may be a promising option to differentiate JGCT among renal mass. The use of a renin inhibitor, which is beneficial in easing symptoms before surgical procedures,^16^ can be facilitated by preoperative diagnosis of JGCT.

## Conflict of interest

The authors declare no conflict of interest.
